# Supplementary material for: Collision risk of bats with small wind turbines: Worst-case scenarios near roosts, commuting and hunting structures
Source: PLoS One. 2021 Jun 25;16(6):e0253782. doi: 10.1371/journal.pone.0253782 (PMC8232403; doi:10.1371/journal.pone.0253782)
Supplement: S1 Table — * = Experiment stopped ahead of schedule due to bat collision. (DOCX) [file pone.0253782.s001.docx]

Appendix 1: Overview over the different operational modes of the turbine applied to each site. * Experiment stopped ahead of schedule due to bat collision

| Date | Site | Start time | End time | Setting |
| --- | --- | --- | --- | --- |
| 28.05.2018 | a | 20:45 | 23:45 | Reference pole |
| 29.05.2018 | a | 20:45 | 23:45 | SWT out of operation |
| 30.05.2018 | a | 21:55 | 00:45 | SWT full motor power |
| 01.06.2018 | a | 20:50 | 23:50 | SWT full motor power |
| 02.06.2018 | a | 20:53 | 23:53 | SWT half motor power |
| 04.06.2018 | b | 21:00 | 00:00 | Reference pole |
| 05.06.2018 | b | 20:54 | 23:59 | SWT full motor power |
| 07.06.2018 | b | 20:58 | 23:59 | SWT out of operation |
| 08.06.2018 | b | 20:56 | 23:54 | SWT half motor power |
| 11.06.2018 | c | 20:58 | 23:58 | Reference pole |
| 13.06.2018 | c | 21:07 | 00:06 | SWT out of operation |
| 14.06.2018 | c | 21:00 | 00:05 | SWT full motor power |
| 15.06.2018 | c | 21:02 | 00:07 | SWT half motor power |
| 16.06.2018 | c | 21:00 | 00:03 | SWT full motor power |
| 18.06.2018 | d | 21:01 | 00:09 | Reference pole |
| 19.06.2018 | d | 20:54 | 23:53 | SWT full motor power |
| 20.06.2018 | d | 20:57 | 00:04 | SWT out of operation |
| 21.06.2018 | d | 21:00 | 22:40 | SWT full motor power |
| 21.06.2018 | d | 22:40 | 00:06 | SWT out of operation |
| 22.06.2018 | d | 20:55 | 00:07 | SWT half motor power |
| 06.08.2018 | e | 20:30 | 23:33 | Reference pole |
| 07.08.2018 | e | 20:30 | 23:30 | SWT out of operation |
| 08.08.2018 | e | 20:30 | 23:36 | SWT full motor power |
| 11.08.2018 | e | 20:30 | 23:20* | SWT half motor power |
| 13.08.2018 | f | 20:15 | 23:18 | Reference pole |
| 14.08.2018 | f | 20:18 | 23:22 | SWT full motor power |
| 15.08.2018 | f | 20:20 | 23:20 | SWT out of operation |
| 16.08.2018 | f | 20:11 | 21:33 | SWT half motor power |
| 17.08.2018 | f | 20:13 | 22:02 | SWT half motor power |
